# The behavioural effects of the serotonin 1A receptor agonist buspirone on cognition and emotional processing in healthy volunteers

**DOI:** 10.1007/s00213-025-06770-6

**Published:** 2025-03-14

**Authors:** Alexander L. W. Smith, Sorcha Hamilton, Susannah E. Murphy, Philip J. Cowen, Catherine J. Harmer

**Affiliations:** 1 Department of Psychiatry, Warneford Hospital, University of Oxford, Oxford, OX3 7JX UK; 2 Oxford Health NHS Foundation Trust, Oxford, UK

**Keywords:** 5-HT, Serotonin, Depression, Buspirone, Cognition, Emotion

## Abstract

**Rationale:**

The 5-HT_1A_ receptor is expressed widely across the brain and is implicated in the mechanism of action of several therapeutics for mood disorders. However, there is limited and contradictory evidence about the role of this receptor in emotional processing and cognition.

**Objectives:**

The current study tested the acute effects of a single dose of the 5-HT_1A_ agonist buspirone (20 mg), on a range of emotional processing (Emotional Test Battery) and cognitive (Auditory Verbal Learning Task (AVLT) and N-back) tasks in healthy, male and female volunteers (N = 62). The study was a randomised, double-blind, placebo controlled, parallel group design.

**Results:**

Buspirone reduced accuracy for detection of facial expressions of disgust and increased misclassification of negative facial emotions. It had no significant effects on categorisation or recall of emotionally-valanced words. Buspirone also reduced recall accuracy in the AVLT but had no significant effect in the N-back task. Participants receiving buspirone were more likely to experience nausea, light-headedness and sleepiness.

**Conclusions:**

Acute buspirone administration produced a mild impairment in verbal memory and a subtle negative bias in emotional processing in healthy volunteers. These effects are consistent with the mixed effects of buspirone on pre- and post-synaptic 5-HT_1A_ receptors.

**Supplementary information:**

The online version contains supplementary material available at 10.1007/s00213-025-06770-6.

## Introduction

The central serotonergic system projects widely throughout the brain and is functionally implicated in key aspects of mood, emotion and cognition. A critical node of control of the serotonergic system is the 5-HT_1A_ receptor, which has complex pharmacology and effects. For example, presynaptic 5-HT_1A_ agonism at 5-HT_1A_ autoreceptors results in a reduction in serotonergic transmission via inhibition of 5-HT release in the terminal fields of serotonergic neurones. However, activation of post-synaptic 5-HT_1A_ receptors can modulate downstream GABA, glutamatergic and cholinergic transmission (Meneses and Perez-Garcia 2007; Ślifirski, Król, and Turło 2021; Ogren et al. 2008). It is through these multifaceted mechanisms that 5-HT_1A_ receptor modulation is implicated in the pathophysiology and treatment of depression (Smith et al. 2023; Sharp and Barnes 2020; Savitz et al. 2009).

5-HT_1A_ receptors influence several brain regions relevant to cognition and emotional processing (Yasuno et al. 2003; Selvaraj et al. 2018), which are often implicated in depression (Sheline et al. 2001; Tartt et al. 2022). Various tasks examining these cognitive and emotional domains, such as N-back and facial expression recognition tasks, have demonstrated impairments in untreated individuals diagnosed with depression (Nikolin et al. 2021; Prado et al. 2018; Talarowska et al. 2010; Harmer et al. 2009), while drugs that have clinical efficacy in Major Depressive Disorder (MDD), typically improve performance on these tasks (Prado et al. 2018; Harmer et al. 2009).

There is a paucity of information on the effects of drugs with 5HT_1A_ receptor agonist properties on emotional processing. A study using a single dose of the partial 5-HT_1A_ agonist, buspirone, reported reduced accuracy in the recognition of fearful (but not happy) faces (Bernasconi et al. 2015). Interestingly, mixed fMRI/PET studies have reported a negative correlation between dorsal raphe (DRN) 5-HT_1A_ binding and amygdala activity to negatively-valanced faces, suggesting that increased activation of DRN 5-HT_1A_ receptors may inhibit amygdala reactivity, and thus blunt neural responses to negatively-valanced emotional stimuli (Fisher et al. 2006; Selvaraj et al. 2015). Taken together, this evidence suggests that buspirone, through presynaptic 5-HT_1A_ agonism, could reduce sensitivity to negatively valanced stimuli.

Studies examining the effects of 5HT_1A_ receptor agonism on non-emotional cognition are inconsistent. Several studies report no impairing effect of buspirone or other 5-HT_1A_ agonists on verbal and working memory performance (Chamberlain et al. 2007; Barbee et al. 1991; Takahashi et al. 2010); however some smaller investigations, which combine cognitive assessment with neuroimaging, do find verbal memory impairment following buspirone administration (Grasby et al. 1992; Yasuno et al. 2003). This inconsistency of results could be attributed to the heterogenous nature of the studies, with some using small sample sizes and male-only participants, or to the use of different measures of memory performance (which vary for example in the number of trials, the inclusion of a reminder of forgotten words between trials and intermediary tasks between trials).

Buspirone is a readily available pharmacological probe for 5-HT_1A_ agonism in humans, however its complex pharmacology should be noted. Acutely buspirone demonstrates full agonism at the inhibitory 5-HT_1A_ receptors, located in presynaptic sites on serotonergic neurons in the raphe nuclei. In essence this action acutely attenuates serotonergic transmission to several brain regions served by the ascending pathways from the raphe such as the prefrontal cortex, hippocampus and amygdala (Vertes and Linley 2007; Artigas 2013). Additionally, buspirone has partial agonism at post-synaptic 5-HT_1A_ receptor sites throughout the brain, with lower efficacy than endogenous 5-HT. Thus the effect of the partial 5-HT_1A_ agonism of buspirone at post-synaptic 5-HT_1A_ receptors may be contextual, with some proposing it has antagonistic action in hyper-serotonergic states and agonist action in hypo-serotonergic states (Yocca 1990). In healthy participants, acute administration of buspirone reliably increases plasma levels of cortisol and growth hormone, indicating agonist action at post-synaptic 5-HT_1A_ receptors in the hypothalamus (Cowen et al 1990).

Furthermore buspirone undergoes marked first pass metabolism to 1-(2-pyrimidinyl)-piperazine, a compound that blocks noradrenergic α_2_-adrenoceptors (Cowen et al. 1990). α_2_-adrenoceptors have been implicated in regulating several neurotransmitter systems (Langer 2015), particularly noradrenaline transmission, which influences working memory (Berridge and Spencer 2016). Furthermore, buspirone apparently has antagonistic activity at both pre- and post-synaptic D_2_ receptors (Loane and Politis 2012).

Here we test the effects of a single 20 mg dose of the 5-HT_1A_ receptor agonist buspirone on emotional processing and working and verbal memory in a large sample of healthy male and female volunteers. We use a battery of emotional processing tasks that are known to be sensitive to the effects of conventional antidepressants (Harmer et al. 2003a, b), as well as testing verbal and working memory using previously used and widely available cognitive tasks (Prado et al. 2018).

We hypothesized that acute administration of buspirone would reduce accuracy for detection of emotional facial expressions, particularly those of negative valance and would have no effect, or a mild impairing effect, on verbal and working memory in healthy volunteers.

## Methods

### Subjects

Sixty-three healthy participants were recruited (28 male, 35 female), aged between 18–50 years through local advertising in community and educational establishments and social media advertising (Meta). Participants were screened for any contraindications to buspirone use. Other exclusion criteria included: previous or current mental illness; previous dependence or recent use of illicit drugs; current pregnancy or breastfeeding; prior completion of tasks used in the study; lactose intolerance and current use of psychoactive medication or medication likely to influence safe participation in the study, which included serotonergic medications or supplements, as judged by a medically-qualified study member. Final inclusion and exclusion criteria are reported in supplementary material. The study was approved by Local Research Ethics Committee (Oxford, MSD-IDREC reference R79236/RE006). Written informed consent was obtained from all participants.

### Design

The study had a double-blind, placebo controlled, between-subject design. Participants were randomly allocated to either placebo or buspirone, stratified by gender. A single buspirone dose of 20 mg was selected based on a balance of target engagement and tolerability (Bernasconi et al. 2015; Cowen et al. 1994; McAllister-Williams and Massey 2003). A single dose of buspirone was chosen, as opposed to repeated dosing, as a single buspirone dose has been shown to produce effective functional activation of pre- and post-synaptic 5-HT_1A_ in healthy participants (Cowen et al. 1994). Participants received either encapsulated placebo (lactose) or buspirone (20 mg). Participants began testing 1 h post capsule administration as this is peak plasma concentration of buspirone (Mahmood and Sahajwalla 1999). Recruitment took place between May 2022-April 2023 in the Department of Psychiatry, Warneford hospital, Oxford. The sample size was calculated based upon the 0.71 effect size of buspirone in depression (Kishi et al. 2014) for 90% power and an alpha 0.05 required 28 per group. This was increased to 31 per group to accommodate attrition.

### Cortisol & temperature measurement

To evaluate engagement of the 5-HT_1A_ receptor, salivary cortisol (collected using Salivette® Cortisol, Sarstedt AG & Co.) and temperature measurements were taken at 30 min intervals; these are recognised surrogate markers of post-synaptic and pre-synaptic 5-HT_1A_ activation respectively (Smith et al. 2023). This gave a total of 8 cortisol and temperature measurements per participant. Saliva samples were rendered acellular and frozen on the day of collection. At the end of participant recruitment, saliva samples were assayed for cortisol using the salivary cortisol enzyme immunoassay kit from Salimetrics, LLC. Temperature was measured from the forehead using a non-contact infrared thermometer (Shenzen Pacom medical instruments).

### Questionnaire and mood & side effects measures

Participants completed the following questionnaires at baseline: Beck Depression Inventory (Beck et al. 1996). Relative to capsule administration, side effects and subjective mood were measured at −30 min (baseline), + 1 h (start of testing) and + 4 h (end of testing) (Supplementary Fig. 1). Participants were required to rate their experience of side effects (nausea, light-headedness, restless and drowsy) and mood (happy, calm, energetic) on a visual analogue scale of 0–100 (not at all – very much). A composite score of side effects and mood was created for each participant at each timepoint e.g. side effects score at 1 h = nausea + light-headedness + restless + drowsiness scores at 1 h.

### Neuropsychological assessment

Participants completed tasks in the same order. Testing was divided into 30 min windows with temperature and salivary samples were taken at the start of each window and a task commenced afterward. The experimenter would leave the testing room when not required for the task. Participants were requested to remain in the room and relax in the time between one task ending and the next window commencing.

#### Emotional processing tasks

The tasks used form part of the Emotional Test Battery (Harmer et al. 2009) which examines the processing of a variety of emotionally valanced stimuli through the use of computerised tasks. Here three tasks were used: Facial Expression Recognition Task (FERT), Emotional Categorisation Task (ECAT) and the Emotional Recall Task (EREC).

During the FERT participants were sequentially shown a human face on a computer screen for 500 ms displaying one of seven emotions (anger, sad, disgust, fear, surprise, happy, neutral) and then replaced by a blank screen (four blocks of either 62 or 63 trials, totalling 250 trials). Emotions were presented in a randomised order and expressed at a range of intensities, morphed between neutral (0%) and full emotions (100%) in 10% steps. Participants were required to report the emotion of the face via a button press as quickly as they could. The task was identical for every participant and took 15 min to complete. Outcome measures were overall % accuracy (when classified by emotion, emotion x intensity and valence) & reaction time. Furthermore, the number of misclassifications of emotions was measured, either broadly e.g. disgust to any other emotion; specifically e.g. disgust to surprise, or by emotion valence e.g. any negative to any positive emotion. The outcome measure for misclassifications was the raw number of trials misclassified for broad and specific misclassifications and percentage of trials misclassified for valence analysis (because subjects underwent differing numbers of trials for each valence e.g. 160 positive and 80 negative).

In the ECAT, participants were shown a series of 40 descriptive personality words (20 positively and 20 negatively valanced), and asked to rate whether would like or dislike being described as each descriptor e.g. empathetic. Words were displayed randomly on a black computer screen for 500 ms and then replaced by a blank computer screen. The task took approximately 13 min to complete. The primary outcome measure was accuracy and reaction time.

The EREC was a surprise free recall task that required the participant to write as many words from the ECAT task (completed approximately 40 min previously) in 4 min. The primary outcome measure was the number of words correctly recalled.

#### Rey auditory verbal learning task (AVLT)

Participants listened to a recording of 15 words (List A) and were asked to immediately recall the list verbally. When the participants were satisfied they had recalled as many words as they could remember, the process was repeated a further four times, with the same recorded words being played before each recall attempt. Participants then listened to a separate, unrelated list of 15 words (List B) and asked to recall them verbally. Immediately after this (short delay) participants were asked to verbally recall as many words from List A as they could remember. This section of the task took approximately 15 min to complete. After a 15-min delay (long delay), participants were again asked to verbally recall as many words from List A as they could remember. Finally, participants were read a list of words (containing the 15 words from List A and 35 distractor words) and were asked to indicate if a word was from list A or not (recognition). The primary outcome measure was number of words correctly recalled.

#### N-back

In this task, adapted from (Mannie et al. 2010), participants were required to indicate (‘*same*’ or ‘*different*’) whether a letter presented on screen matched the letter presented *n* trials previously (where *n* equals one, two, three, representing 1-back, 2-back or 3-back respectively). Letters were presented for 500 ms, with a fixation cross presented for 1500 ms between letters. Participants underwent 160 trials in total (40 trials per condition) in blocks of 10 trials, a fixation cross was presented for 5000 ms between each block. Each block was for one condition only e.g. all 1-back or all 2-back). The task took approximately 20 min to complete. Outcome measures were overall accuracy and average reaction time for each condition for each participant.

### Statistical analysis

All analyses were performed using R software (version 2023.09.1). A repeated measure analysis of variance (ANOVA) was performed to evaluate changes in mood and side effect burden between groups during the study, with individual rating, allocation group and timepoint as predictor variables. Additionally, composite scores for each category of rating was taken e.g. mood = calm + energetic + happy and compared between groups at the different timepoints.

The mean average cortisol measurement was calculated for each allocation group at each timepoint. The difference in temperature from baseline at an individual level was calculated. A mean average of the difference from baseline was calculated for each allocation group at each timepoint. Side effect score was calculated as a composite score of all side effect ratings (nausea, restless, sleepy and light headedness) at the start of testing (e.g. 1 h).

Behavioural data were analysed using a repeated measures ANOVA. Group allocation (buspirone or placebo) was used as a between subjects’ factor in all tests. For FERT analysis the seven emotions were further classified into valence categories that were negative (anger, sad, disgust, fear), positive (surprise, happy) or neutral (neutral). Emotion, intensity and valence were then used as predictor variables for the FERT task in separate analyses. To investigate misclassification of emotions, the number of misclassifications was used as an outcome variable. Three separate approaches were used to analyses misclassifications. These were for broad misclassifications (an emotion misclassified to any other emotion, e.g. anger misclassified to happy, sad, disgust etc.), specific misclassification (an emotion misclassified to a specific emotion, e.g. anger misclassified to sad) and valence (a positive, negative or neutral emotion misclassified to an emotion of different valence or another emotion within the same valence e.g. a positive emotion misclassified to negative emotion). Significant results were followed up with post hoc t-tests (Bonferroni corrected).

In the EREC and ECAT analysis, word valence was a predictor variable and total number of words recalled an outcome variable.

The outcome variable for the AVLT was number of words recalled in the first five free recall trials and the delayed free recall (short & long) trials. Acquisition block (AVLT) and condition (N-back) were additional predictor variables.

A composite side effect score at 1 h was used for sensitivity analysis, as deemed the most relevant when evaluating the impact of side effect score on behavioural testing.

## Results

### Missing data and outlier removal

One participant was excluded from all analyses having received a half dose of the study medication in error (buspirone group).

All outlier removal, based upon accuracy and reaction time, was performed prior to unblinding. In analyses of FERT data, all data from one participant was excluded from the final FERT analyses as they were more than two standard deviations outside of the mean for accuracy for the entire dataset. This resulted in 31 participants in the placebo group and 30 participants in the buspirone group.

For the aforementioned tasks less than 200 ms was chosen as a lower cut off as responses below this time are deemed too fast to register stimuli and enact a motor response. This resulted in removal of 1 trial in the FERT and 1 trial in the ECAT. Across both tasks, if a participant’s performance met the criteria as an extreme outlier (trials lying at more than three times the participants’ interquartile range above their third quartile), it was removed, as per previous analysis of the tasks (Murphy et al. 2020). This resulted in removal 311 trials (2%) in the FERT and 44 trials (1.8%) in the ECAT.

In analysis of N-back data, the data of one participant was removed due to consistently being more than two standard deviations outside of the mean for accuracy, resulting in 31 participants in the placebo group and 30 participants in the buspirone group.

Three participants (all buspirone) were excluded from EREC analysis due to errors in data collection, leaving 29 participants in the buspirone group and 31 participants in the placebo group.

No participants or trials were excluded from the AVLT analysis.

Two participants (both in buspirone allocation group) were omitted from side effect analysis and subsequent sensitivity analysis due to an error in collection of side effect data.

### Demographics, mood and side effects

The groups were well matched for age, gender and baseline mood (Supplementary Table 1). There was a main effect of group on mood ratings [F(1, 50) = 5.84, p = 0.019), which reflected lower composite mood ratings (calm, energy and happy) in the buspirone group (t(50) = −2.39, p = 0.021). Specifically at 1-h post-intervention the buspirone group, compared to the placebo group, felt less happy (t(54) = −2.71, p = 0.009) and less energetic (t(58) = −4.82, p < 0.001). There was a significant main effect of group on the side effect ratings [F(1, 59) = 13.32, p < 0.001]. There was also a significant interaction between specific side effect, timepoint and group [F(6,659) = 3.01, p = 0.0065). Post hoc testing indicated that at 1 h post-intervention the buspirone group, compared to the placebo group, experienced more nausea (t(31) = 2.26, p = 0.031), light-headedness (t(31) = 4.67, p < 0.001) and sleepiness (t(35) = 2.97, p = 0.0054). These group differences were non-significant by the end of testing (Supplementary Table 2).

### Cortisol & temperature measurement

**Fig. 1:**
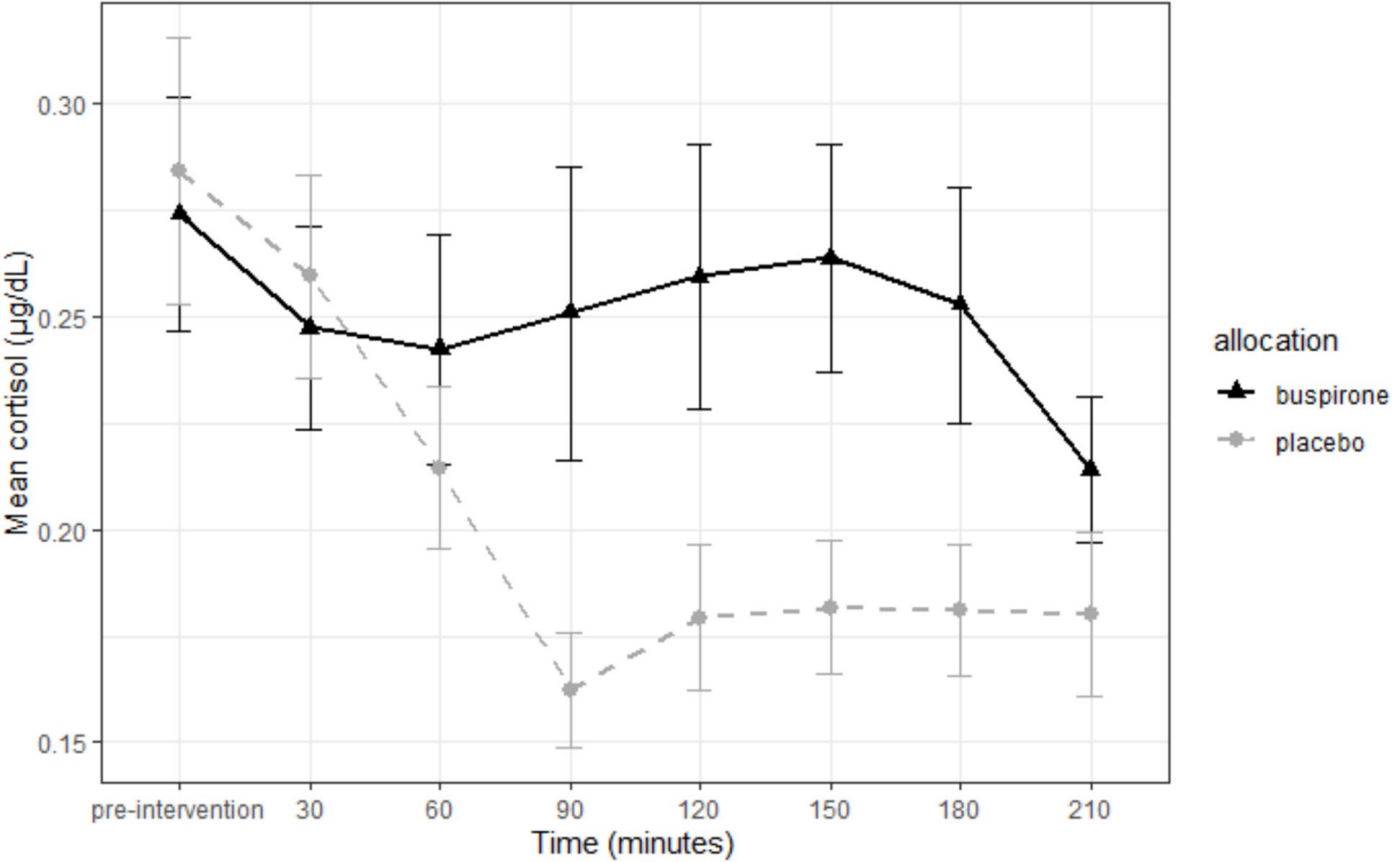
Mean salivary cortisol response following either buspirone or placebo. Error bars = ± S.E.M

There was a significant interaction between group and timepoint for cortisol measurement [F(7, 427) = 2.54, p = 0.014]. Figure 1 illustrates the buspirone group maintained salivary cortisol levels whilst the placebo group showed lowered cortisol levels during the testing period. The difference in cortisol between groups became significant by 90 min post-intervention (t(40) = 2.39, p = 0.021), with the placebo group showing diminished cortisol levels. This difference remained until 3 ½ hours post intervention. This could represent engagement of post-synaptic hypothalamic 5-HT_1A_ receptors by buspirone (Cowen et al. 1990).

No significant effect of time (F(4,54) = 1.02, p = 0.406) or allocation (F(1,54) = 2.09, p = 0.154) was observed for temperature. However, visual inspection of change in temperature from baseline (Supplementary Fig. 2) indicated a difference between allocation groups at certain timepoints, such as a non-significant difference between groups at time 150 min (t(57) = −1.8, p = 0.077).

### Emotional test battery

#### Facial expression recognition test (FERT)

##### Accuracy

**Fig. 2:**
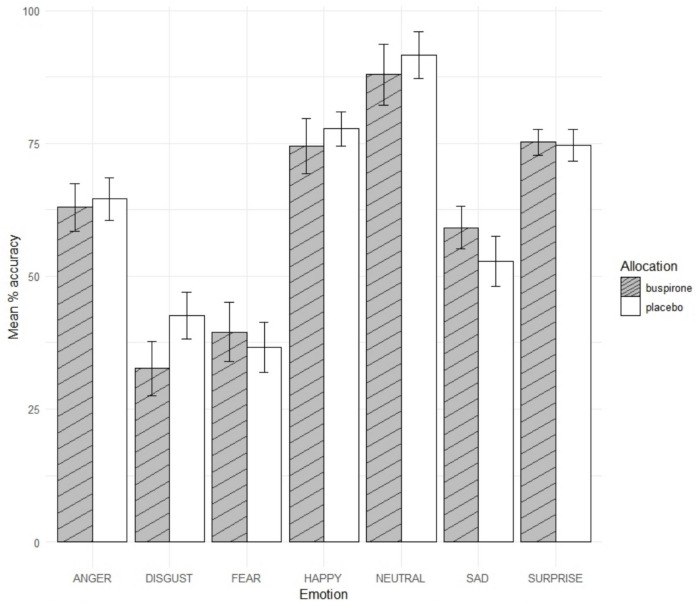
Mean percentage accuracy for emotional categories in FERT. Error bars = 95% C.I

There was a significant group by emotion interaction [F(6,322) = 3.14, p = 0.005, η^2^_p_ = 0.055) on accuracy of facial expression recognition (Fig. 2). Further analyses indicated that the buspirone group were less accurate in recognising disgust [t(56) = −2.89, p = 0.005, d = −0.75, CI = −16.88 to −3.07, buspirone = 32.63, placebo = 42.61] and more accurate in recognising sad emotions [t(57) = 2.01, p = 0.049, d = 0.47, CI = 0.023 to 12.57, buspirone = 59.11, placebo = 52.81] (Fig. 2). There was no significant main effect of group [F(1, 50) = 0.034, p = 0.56] or group by emotion interaction [F(6, 322) = 1.86, p = 0.088] for reaction time.

A non-significant main effect of allocation was observed for accuracy when including intensity of emotion in testing (F(1,59) = 0.56, p = 0.46, η^2^_p_ = 0.056).

When facial emotions were collapsed into valence categories (positive, neutral, negative), there was no significant main effect of group on accuracy [F(1,59) = 1.61, p = 0.21] or reaction time [F(1,59) = 0.015, p = 0.90]. There was no significant interaction effect between group and valence for accuracy [F(2, 118) = 0.32, p = 0.73) or reaction time [F(2, 118) = 0.84, p = 0.44).

##### Misclassification

Group allocation did not have a significant main effect on the total number of broad misclassifications (F[1, 48] = 0.61, p = 0.44), however significant interaction between group and emotion was observed (F[3, 48] = 3.00, p = 0.040, η^2^_p_ = 0.16), which was possibly driven by a greater number of misclassifications of anger in the buspirone group i.e. reporting more angry faces than were present (t(43) = 1.91, p = 0.063, 95% CI =—0.09 to 3.19, buspirone = 6.55; placebo = 5.00, Cohen’s d = 0. 5).

There was a significant interaction between group and specific misclassification [F(41, 2419) = 1.64, p = 0.0069, η^2^_p_ = 0.027). This was driven by significantly increased misclassification of anger to sad [t(42) = 2.29, p = 0.027, 95% CI = 0.17 to 2.65, Buspirone = 2.7, placebo = 1.3, Cohen’s d = 0.59)] and happy to fear [t(31) = 2.24, p = 0.032, 95% CI = 0.11 to 2.31, buspirone = 1.57, placebo = 0.35, Cohen’s d = 0.58] and significantly decreased misclassification of fear to neutral [t(56) = −2.37, p = 0.021, 95% CI = −2.76 to −0.23, buspirone = 9.53, placebo = 11.03, Cohen’s d = −0.60)]. Broadly this could be interpreted as acute buspirone maintaining, or possibly inducing, a negative bias in emotional processing, compared to placebo.

**Fig. 3:**
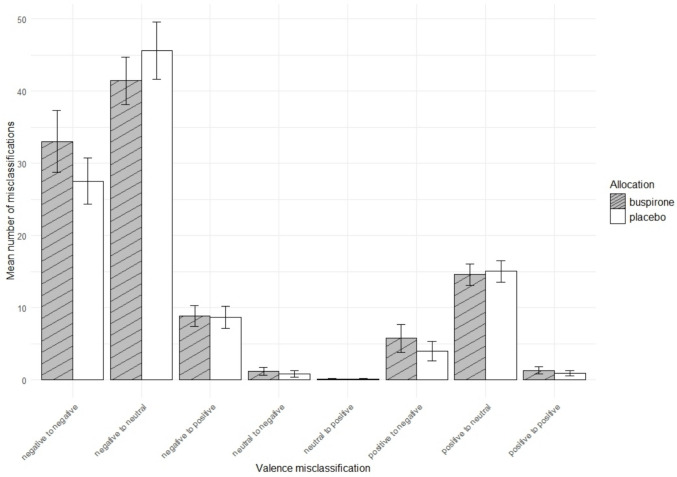
Mean number of misclassifications by valence in the FERT. Error bars = 95% C.I

When the face emotions were collapsed into valence categories (positive, neutral, negative) there was a significant group x valance misclassification interaction [F(7, 413) = 2.98, p = 0.005, η^2^_p_ = 0.048) (Fig. 3). This was possibly driven by a significantly increase in misclassification of negative to negative e.g. sad to anger or disgust to sad [t(54) = 2.03, p = 0.047; 95% CI = 0.031 to 4.43, buspirone = 13.2%, placebo = 11.0%, Cohen’s d = 0.52], supporting the interpretation that acute buspirone may maintain, or possibly induce, a negative bias in emotional processing.

#### Emotional categorisation words

There was no significant effect of group on the percentage of words correctly classified (F[1, 60] = 0.00, p = 0.99) or on the average reaction time to words (F[1, 60] = 0.20, p = 0.66).

#### Emotional word recall task

There was no significant effect of group on accurate recall of positive or negative words F[1, 60] = 0.099, p = 0.75).

### Cognitive testing

#### AVLT

**Fig. 4:**
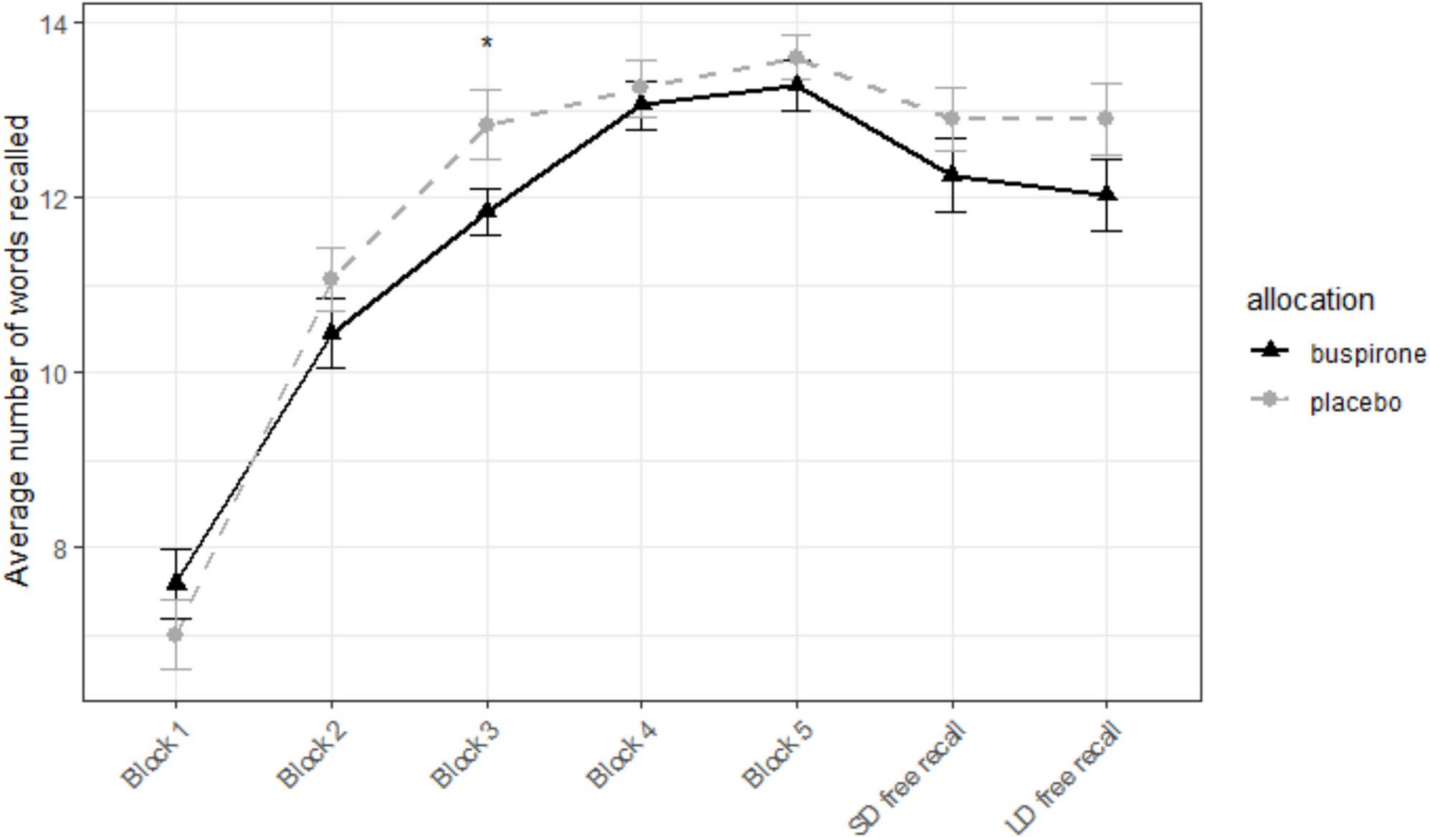
Performance on the AVLT. Mean number of words recalled by the placebo and buspirone groups immediately following presentation of the word list (five repetitions) and following a short delay (SD free recall) and long delay (LD free recall). Asterisks represent significant level of difference between groups *p < 0.05. Error bars = ± S.E.M

No significant effect of group was found on average number of words recalled during all free recall trials (t(60) = −1.05, p = 0.3). There was a significant block by group interaction [F(6, 360) = 2.76, p = 0.012, η^2^_p_ = 0.044) on the AVLT, which reflected poorer recall in the buspirone group compared to placebo. This group difference was significant on block 3 (t(52) =—2.06, p = 0.044, 95% CI = −1.97 to −0.028, buspirone = 11.84, placebo = 12.84, Cohen’s d = −0.52). There was no significant group difference in the long delay free recall (t(60) = −1.47, p = 0.15) and long delay recognition (t(48) = −1.10, p = 0.28) blocks, although the buspirone group again had numerically poorer performance (Fig. 4). There was no significant difference in recall of the distractor word list B (t(57) = 0, p = 1).

#### N back

**Fig. 5:**
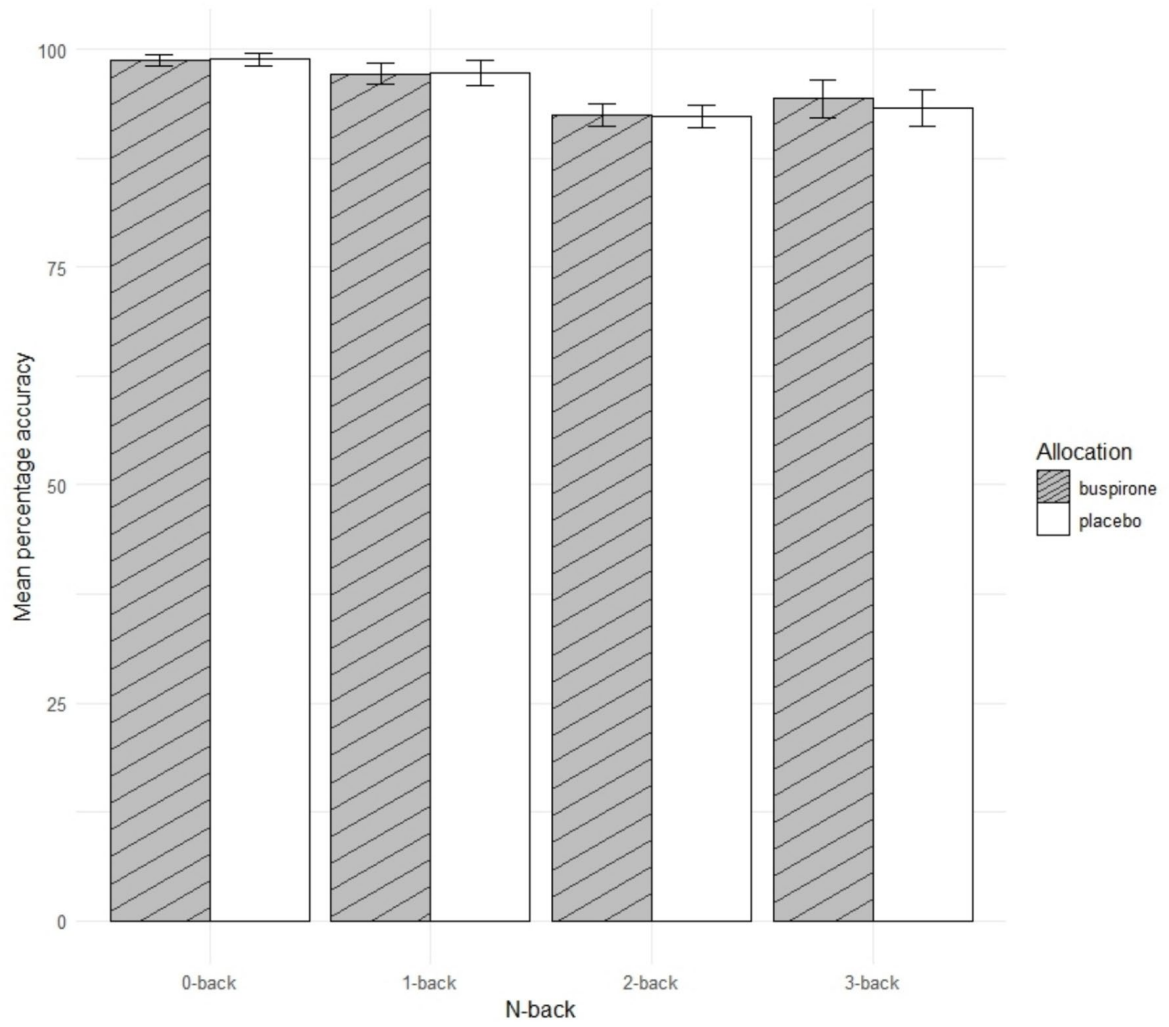
Mean percentage accuracy for each N-back category in the N-back task. Error bars = 95% C.I

There was no significant main effect of group allocation on overall accuracy [F(1,59) = 0.16, p = 0.69) or average reaction time [F(1,59) = 0.00, p = 0.97) (Fig. 5). Furthermore there was no interaction between group allocation and condition for overall accuracy [F(3,177) = 0.39, p = 0.76) or average reaction time [F(3,177) = 0.83, p = 0.48).

### Sensitivity analysis: Side effect as a covariate

The analyses were repeated with the inclusion of the composite side effect score (one-hour post drug administration) as a covariate in order to investigate whether any of the observed effects were driven by the different side effects experienced by the two groups. Despite a group difference in side effect scores, no statistically significant main effect of side effect was found in subsequent sensitivity analysis. To explore the effect further Pearson’s correlation coefficients were calculated for outcome measures and the composite side effect score and mood score separately.

#### Facial expression recognition test (FERT) – accuracy

The significant group by emotion interaction on accuracy of facial expression recognition in the FERT remained when including side effect score as a covariate (F(6, 311) = 2.74, p = 0.013, η^2^_p_ = 0.050). There was a positive correlation between accuracy for happy faces and the composite mood score, r(36) = 0.36, p = 0.026. No other correlations were significant.

#### Facial expression recognition test (FERT) – misclassifications

When including side effect score as a covariate the significant interactions remained between group and emotion for broad misclassifications [F(3,47) = 3.13, p = 0.034, η^2^_p_ = 0.16], between group and emotion of specific misclassification (F(41, 2337) = 1.45, p = 0.034, η^2^_p_ = 0.025) and between group and valance of misclassification interaction [F(7, 413) = 2.98, p = 0.005, η^2^_p_ = 0.047]. A positive correlation arose only for percentage of anger misclassifications and composite side effect score, r(55) = 0.27, p = 0.046. Examining specific misclassification, the number of anger to sad misclassifications negatively correlated with mood scores (r(53) = −0.33, p = 0.013) and positively correlated with side effect scores (r(57) = 0.30, p = 0.02). Misclassification of sad to anger also positively correlated with side effects core at time of testing (r(57) = 0.36, p = 0.0049). Misclassification of happy to fear positively correlated with mood scores as well (r(53) = −0.45, p < 0.001). No significant correlations arose between valence and composite scores.

#### AVLT

The significant interaction of group and block remained in the AVLT when side effect score was included as a covariate (F(6, 348) = 2.60, p = 0.018 η^2^_p_ = 0.042). No significant correlation arose between number of words successfully recalled at each trial stage and the composite mood or side effect score.

## Discussion

### Summary of results

This study broadens the evidence for the effects of acute 5-HT_1A_ receptor agonism in cognition and emotional processing. Specifically, in a healthy volunteer sample, we found that buspirone reduced sensitivity to disgust emotions, increased sensitivity to sad emotions and increased misclassifications of negative emotions to other negative emotions. We found no effect of buspirone on working memory performance but a subtle impairment in verbal memory.

### Emotional processing

Our findings demonstrate that acute buspirone administration increases negative to negative emotion misclassifications compared to placebo. This could indicate that buspirone induces a subtle negative bias in emotional processing perhaps through the reduction of serotonergic transmission that follows acute activation of 5-HT_1A_ autoreceptors. In support of this, it has been reported that a reduction in serotonergic transmission, through depletion of the 5-HT precursor tryptophan, induces a negative affective bias in healthy participants and remitted depressed patients (Hayward et al. 2005; van der Veen et al. 2007). Consistent with this acute tryptophan depletion (ATD) attenuated a positive bias in an affective word task (Roiser et al. 2008) and an increase in 5-HT transporter binding potential has been linked to a negative affective bias in healthy volunteers (Armand et al. 2022). Broadly it seems a reduction in serotonergic transmission leads to a negative affective bias behaviourally. However, it is noteworthy that several studies have observed no effect of ATD on performance, albeit with incidental, gender identification tasks rather than explicit emotional recognition tasks as used here (Daly et al. 2010; Fusar-Poli et al. 2007). A common finding of all studies though is reduced serotonergic transmission being associated with increased and decreased neural responses to negative and positive emotional stimuli. These have been with accompanying behavioural changes in emotional-valanced verbal stimuli (Roiser et al. 2008) and a facial emotion perception task (van der Veen et al. 2007) and without accompanying behavioural changes (Fusar-Poli et al. 2007; Daly et al. 2010). Studies have implicated similar regions in responses to emotionally-valanced tasks such as the cingulate cortex and insula (Roiser et al. 2008; Daly et al. 2010) as well as regions commonly associated with emotional processing such as the amygdala (van der Veen et al. 2007). Others identifying increased amygdala activity in emotionally valanced tasks under ATD conditions, also found a decrease in prefrontal-amygdala connectivity when viewing negative vs positive emotions, a so-called ‘aversive amplification circuit’ (Robinson et al. 2013). It is possible, therefore, that reduced serotonergic transmission from acute buspirone administration could lead to disinhibition of a prefrontal – amygdala aversive amplification circuit and increased sensitivity to negative emotions, as observed in results presented here.

However, ATD and buspirone are not equivalent in terms of pharmacological effects, because buspirone’s post-synaptic actions may mitigate the behavioural effects of a net reduction in serotonergic transmission brought about from buspirone-induced 5-HT_1A_ autoreceptor activation. Namely buspirone is known to possess partial agonism activity at post-synaptic 5-HT_1A_ heteroreceptors (Bantick et al. 2004), many of which are found in regions implicated in emotional processing (Puig and Gulledge 2011; Barnes and Sharp 1999). As such, the partial agonism activity at 5-HT_1A_ heteroreceptor of buspirone may maintain 5-HT_1A_-related inhibition of these regions, which would not be the case with the broad decrease in 5-HT neurotransmission produced by ATD. Speculatively acute buspirone may result in a partial disinhibition of the prefrontal-amygdala circuit, thereby producing a minor increase in sensitivity to negative emotions.

Our observation of changes in negative bias following buspirone is in partial agreement with the only directly comparable study of 15 healthy volunteers (Bernasconi et al. 2015). This study found buspirone reduced sensitivity to fear when presented for 90 ms only (Bernasconi et al. 2015). Here we find reduced sensitivity to another negative emotion, disgust but also an increased sensitivity to sad emotions.

Several reasons could account for this discrepancy in results between the two studies. Firstly the task used here presented emotional faces for 500 ms, greater than 90 ms presented in the task used by Bernasconi et al.; this would result in greater cognitive analysis of faces, employing cortical regions such as the prefrontal cortex (Wong et al. 2009). This study therefore extends the work of Bernasconi et al. in indicating that the behavioural influence of buspirone, a 5-HT_1A_ receptor agonist, can have mixed effects on differing emotions of the same valence, potentially through effects in cortical areas. Our study also used a greater number of emotions and required specific labelling of emotions, as opposed to the emotion matching task of either fearful or happy faces, requiring binary responses, used by Bernasconi et al.. As such, our design allowed effects of buspirone on other emotions e.g. disgust and sad, to be measured. However, the fact that no significance difference in accuracy for recognition of fear or sad faces was observed, could be due to the complex nature of the ETB emotion recognition task requiring greater cognitive resource in specific labelling of emotions. Finally, Bernasconi et al. recruited more males than females, compared to the participant group here which had a greater number of females. This may be important because females are more sensitive to negative emotions and emotion processing in general (Thompson and Voyer 2014), perhaps allowing more treatment effects to be detected in the present study potentially.

Accuracy in emotional facial expression recognition has been shown to worsen in individuals diagnosed with depression (Surguladze et al. 2004). Hence change in mood state induced by buspirone could alter accuracy of facial emotion recognition. Whilst buspirone elicited a decrease in subjective mood, this did not correlate with significant differences in accuracy emotion recognition or misclassification. It is also worth noting that with the ETB, depressed patients showed an improvement in recognition of positive emotions, after a single dose of reboxetine, which had no impact on mood ratings (Harmer et al. 2009) i.e. performance on emotional tasks can be improved by pharmacological interventions prior to clinical enhancement in mood, which typically takes days or weeks to develop with conventional agents (Harmer et al. 2003a, b).

### Cognition

The results presented here indicate acute buspirone produced no significant effects on working memory but did elicit a small, deleterious effect on short term, verbal memory. This observation is consistent with some but not all previous buspirone studies. For example, a small, healthy male volunteer study observed that acute administration of buspirone 30 mg negatively affected performance on a verbal memory task (Grasby et al. 1992). This study also reported a reduction in activity following buspirone administration in the retro-splenial cortex (a region linked to the hippocampus), the right prefrontal cortex and the parahippocampal gyrus during performance of the task (Grasby et al. 1992). Our results are also supported by behavioural studies which report a worsening of memory performance, following administration of other 5-HT_1A_ receptor probes such as tandospirone (Yasuno et al. 2003) and ipsapirone (Riedel et al. 2002).

A potential pharmacological mechanism for our results could be found from animal models of cognition, which support the concept of differential effects of 5-HT_1A_ agonism depending on receptor location. Specifically post-synaptic 5-HT_1A_ agonism could impair cognition in humans (King et al. 2008). Therefore, a probe such as buspirone with modest post-synaptic action, could impair cognition via its downstream inhibitory effect on GABA interneurons and glutamatergic pyramidal cells. This would occur in regions relevant to cognition, such as the hippocampus, where a reduction in glutamatergic transmission could lead to an overall impairment in cognitive function.

However, similar to results presented here, several studies have observed buspirone to have no deleterious effect on episodic memory (Barbee et al. 1991; Unrug-Neervoort et al. 1992; Chamberlain et al. 2007). One commonality for all studies is that an aggregate of free recall was used as an outcome measure. Similarly, our analysis using an aggregate score found no difference. However, when separating by trial block (a comparison not performed in many of the aforementioned studies), a difference between groups emerged, indicating an impairment in immediate recall but at an earlier timepoint than is often tested.

### Limitations

Limitations of this work include the use of buspirone as a probe of 5-HT_1A_ receptors, in that it has differing efficacy at pre-and post-synaptic 5-HT_1A_ receptors. Namely, acute buspirone would be expected to reduce 5-HT release in the terminal fields of serotonergic neurones originating from the raphe nucleus, leading to a broad decrease in activation of 5-HT receptors in post-synaptic sites. However the partial agonism of post-synaptic 5-HT_1A_ receptors could maintain the inhibitory influence on brain regions relevant to memory and emotion such as the hippocampus and enterohinal cortex as well as the insula, anterior cingulate, frontal cortex (Ito et al. 1999).

The activity of buspirone’s major metabolite l-(2-Pyrimidinyl)-pipcrazine (1-PP) also complicates interpretation. For example, 1-PP has been shown in vivo to inhibit somatodendritic and terminal α_2_-adrenergic autoreceptors in the rat brain, leading to a disinhibition of the noradrenergic system (Blier et al. 1991). Intriguingly a previous healthy volunteer study using a single dose of the noradrenaline-reuptake inhibitor, reboxetine, had no effect on AVLT but increased positive bias to positive emotional faces, results which are not replicated here (Harmer et al. 2003a, b). One potential explanation for this discrepancy is the noradrenergic actions of 1-PP after a single dose of buspirone are insufficient to induce a net positive shift in emotional processing.

Furthermore, buspirone possess affinity for D_2_ and D_3_ receptors, albeit to a lesser degree than 5-HT_1A_ receptors (Loane and Politis 2012) and with a binding affinity at the dose used in the current study, that is unlikely to yield significant behavioural effects alone (Le Foll et al. 2016). However, this multimodal pharmacology of buspirone could contribute synergistically to widespread effects on cognition and emotional processing, making it challenging to attribute any effects (or lack thereof) to 5-HT_1A_ receptor agonism alone. This question could be addressed by more specific probes, such as the biased 5-HT_1A_ agonist NLX-101 (post-synaptic preference) or NLX-112 (pre-synaptic preference) (Newman-Tancredi et al. 2022; Smith et al. 2023).

As expected, buspirone produced characteristic side effects such as light-headedness and nausea. These did influence the significance of a few results, for instance for the FERT, accuracy and specific misclassifications. However, the majority of significant results within these analyses remained e.g. accuracy for disgust and misclassification of disgust to sad. This indicates side effect burden does not generally account for the results presented here.

This study’s use of cortisol measurements provides a surrogate marker of 5-HT_1A_ post-synaptic target engagement. However, a hypothermic response was not observed; this is surprising as buspirone is know to possess significant presynaptic 5-HT_1A_ receptor activity. This could be due to temperature measurement issues e.g. inconsistency in measurement, thermometer imprecision. In future, alternative means of measurement could be used, such as tympanic thermometers. Alternatively, it could be a feature of the buspirone dose used, as administration of a higher dose buspirone (30 mg) does produce a hypothermic response (Cowen et al. 1990; Young et al. 1993), as does acute challenge with other 5-HT_1A_ receptor agonists such as tandospirone and gepirone (Yasuno et al. 2003; Anderson et al. 1990). A PET-fMRI study with a 5-HT_1A_ agonist, could provide more direct evidence of target engagement.

Another drawback is the number and breadth of cognitive tests used (N-back and AVLT). The number of tests used was balanced against participant fatigue, although it is acknowledged this restriction in number of cognitive tests used possibly limits the cognitive domains explored. In future, additional cognitive domains could be assessed such as executive function (e.g. Wisconsin card sorting task) or processing speed (e.g. Digit Symbol Substitution Test), using widely available tasks.

The use of healthy volunteers may limit the sensitivity of the measures to detect a drug effect, because task performance is already high in this group. As such, examination of clinical samples, such as those diagnosed with schizophrenia or major depressive disorder, may give richer insights into the effects of 5-HT_1A_ manipulation on cognition and emotional processing. Furthermore, the effects of buspirone may be different in groups in which there are pre-existing alterations in the monoaminergic system, such as those with mood disorder.

A final limitation would be the use of between subject comparison which, although improving generalizability of results and using relatively greater numbers than comparable studies, it may be insufficient to detect subtle, individual-level effects that could manifest with a within-subject design (Bernasconi et al. 2015).

### Clinical implications

Given as a sole treatment, acute 5-HT_1A_ receptor agonism appears to induce a subtle negative emotional bias and impairment of verbal working memory, albeit to a degree that may not be clinically meaningful. Broadly, across the world, buspirone alone is not recommended for the treatment of MDD (Taylor et al. 2020). Results presented here, namely of increased sensitivity to sadness and an increase in negative to negative misclassification, would support this. However, caution is required when extrapolating effects of a single buspirone dose to the longer-term period of treatment required in clinical practice. The potential difference produced by extended duration of treatment is evidenced by meta-analytic evidence indicating that 6-week courses of buspirone alone do increase response rates in MDD (Kishi et al. 2014). Furthermore, it highlights a potential differential effect of buspirone between healthy volunteers and a clinical population. Contrasting evidence exists for the sensitivity of 5-HT_1A_ autoreceptors in MDD, some indicating sensitivity is reduced, especially in melancholic subtype (Cowen et al. 1994), whilst others have found an increase in a non-melancholic subtype (Navines et al. 2006). Speculatively, on the premise of increased 5-HT_1A_ autoreceptor sensitivity in non-melancholic depression, it could be that acute buspirone would reduce serotonergic transmission further and thereby maintain a negative emotional bias.

It is of interest that acute buspirone reduces sensitivity to disgust. This could suggest potential value of buspirone in treatment of conditions that involve altered disgust processing, such as obsessive–compulsive disorder. However, trial evidence for this is mixed with some evidence of benefit and some of no benefit with buspirone either alone or as an augmenting agent (Loane and Politis 2012; Garg and Tyagi 2021).

However in the treatment of MDD it is noteworthy that vortioxetine, a medication that combines serotonin reuptake inhibition with 5-HT_1A_ receptor agonism, has beneficial effects on cognition in MDD (Mahableshwarkar et al. 2015; Bennabi et al. 2019) and to a greater extent than treatment with a selective serotonin reuptake inhibitor (SSRI) alone (Sagud et al. 2021).

In terms of emotional processing, vortioxetine reduced emotional blunting in a sample experiencing depression which had partially responded to SSRI medication (Fagiolini et al. 2021), indicating that the addition of 5-HT_1A_ receptor agonism to serotonin reuptake inhibition could have a beneficial effect on both emotional processing and cognition, that neither mechanism would have alone. However, in these vortioxetine studies, 5-HT_1A_ receptor activation would have occurred through an extended period of treatment, which, from pre-clinical work, would be expected to result in desensitisation of 5-HT_1A_ autoreceptors and a freeing of 5-HT neurons from this aspect of inhibitory control (Blier and Ward 2003). This will make it important in future studies to address the cognitive and emotional effects of repeated administration of 5-HT_1A_ receptor agonists in both healthy participants and patients with mood disorders.

## Supplementary information

Below is the link to the electronic supplementary material.

ESM 1 (DOCX 54.0 KB)

## Data Availability

Data available on request.
