# Supplementary material for: The behavioural effects of the serotonin 1A receptor agonist buspirone on cognition and emotional processing in healthy volunteers
Source: Psychopharmacology (Berl). 2025 Mar 14;242(8):1859–73. doi: 10.1007/s00213-025-06770-6 (PMC12296846; doi:10.1007/s00213-025-06770-6)
Supplement: Supplementary file 1 — (DOCX 54.0 KB) [file 213_2025_6770_MOESM1_ESM.docx]

# **The behavioural effects of the serotonin 1A receptor agonist buspirone on cognition and emotional processing in healthy volunteers: Supplementary information**

Alexander L.W. Smith^1,2^;

Sorcha Hamilton^1,2^;

Susannah E. Murphy^1,2^;

Philip J. Cowen^1,2^;

Catherine J. Harmer^1,2^;

^1^ Department of Psychiatry, Warneford Hospital, University of Oxford, Oxford OX3 7JX, UK

^2^ Oxford Health NHS Foundation Trust, Oxford, UK

**Author for correspondence**: Alexander L. W. Smith, E-mail: alexander.smith@psych.ox.ac.uk

**Supplementary text 1.**

Inclusion criteria

- *Participant is willing and able to give informed consent for participation in the research*
- *Male or female*

Exclusion criteria

*The participant may not enter the study if ANY of the following apply:*

- *Any current Axis 1 DSM-5 psychiatric disorder*
- *Any previous episode of a severe mental illness,*
- *A first degree relative diagnosed with Bipolar Affective Disorder Type 1 or Schizophrenia*
- *Body Mass Index less than 17 or deemed unacceptable by the medically-qualified team member on grounds of safety or scientific integrity*
- *Any significant current medical condition likely to interfere with conduct of the study or analysis of data (epilepsy, renal disease, hepatic disease, myasthenia gravis, acute closed-angle glaucoma)*
- *Current use of psychoactive and / or medically significant medication as judged by a medically-qualified team member, whether prescribed or bought over the counter (the contraceptive pill, the Depo-Provera injection or the progesterone implant* *will not result in exclusion)*
- *Past history of dependence to illicit substances, and any consumption of illicit substances in the three months prior to the study*
- *Currently pregnant or breast feeding*
- *Known lactase deficiency or any other problem absorbing lactose, galactose, or glucose*
- *Participation in a study using the same tasks in the last year*
- *Any physical (including visual and auditory) or language impairment that would make complying with the study protocol challenging. This includes any taste/olfactory disturbance e.g. secondary to Covid-19 infection*

**Supplementary Figure 1:** schematic of testing

Saliva sample & temperature recorded every 30 mins.

VAS = visual analogue scales, psychometric questionnaires included Beck Depression Inventory, Apathy Motivation Index, Snaith-Hamilton Pleasure Scale and Temporal Experience of Pleasure Scale

Temperature change

No significant temperature change between groups emerged (F(7, 407) = 0.22, p = 0.14), however an unpaired t tests of temperature change from baseline did reveal a non-significant temperature differences between groups at 2hrs (t(57) = -1.8, p = 0.077, buspirone mean = -0.03^o^C; placebo mean = 0.067^o^C; 95% CI = -0.21 – 0.011).

**Supplementary Figure 2**: Line plot of average change from baseline temperature over time, grouped by allocation group. Error bars = +/- 1 S.E.M.


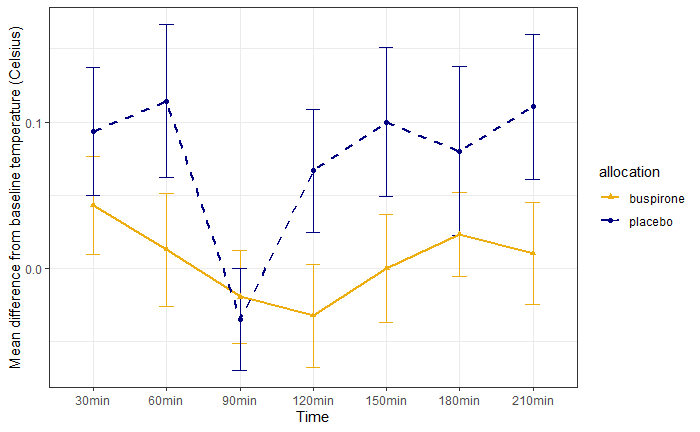


| Supplementary Table 1: Demographics and psychometrics of participants | | |
| --- | --- | --- |
|  | Buspirone (N = 31) | Placebo (N = 31) |
| Gender, N female:male | 17:14 | 17:14 |
| Age (years), mean (S.D.) | 25.19 (6.00) | 27.28 (8.72) |
| BDI^a^, mean (S.D.) | 3.03 (3.03) | 2.19 (2.71) |
| ^a^ BDI = Becks Depression Inventory | | |

**Supplementary Table 2:** Mean scores of mood and side effects ratings, grouped by allocation & timepoint

| Rating | Allocation | Timepoint | | |
| --- | --- | --- | --- | --- |
|  |  | Baseline | 1 hour | 4 hours |
| Happy | Buspirone | 65.7 (18) | 60.4 (19.5) | 68.7 (13.8) |
|  | Placebo | 70.4 (19.1) | 73.6 (17.5) | 75.2 (14.6) |
| Calm | Buspirone | 79.1 (20.1) | 72.5 (21.9) | 80.1 (14.2) |
|  | Placebo | 77.5 (19.2) | 80.9 (14.6) | 77.9 (15.6) |
| Energetic | Buspirone | 59.6 (17.6) | 41.9 (19.9) | 51.8 (17.5) |
|  | Placebo | 63.2 (21.5) | 65.5 (18.4) | 59.4 (24.3) |
| Nausea | Buspirone | 1.9 (5.7) | 5.7 (12) | 3.3 (10.7) |
|  | Placebo | 0.9 (2.8) | 0.7 (2.4) | 0.2 (0.7) |
| Lightheadedness | Buspirone | 4.6 (12.2) | 26.4 (28) | 5.7 (14.4) |
|  | Placebo | 1 (3.6) | 2.2 (4.7) | 1.7 (4.7) |
| Restless | Buspirone | 3.8 (13) | 5.8 (15.5) | 5.5 (13.8) |
|  | Placebo | 4.2 (15.5) | 1.5 (3.5) | 1.9 (4.5) |
| Sleepy | Buspirone | 13.2 (16.5) | 21.7 (26.7) | 14.6 (19.8) |
|  | Placebo | 6.6 (12) | 6.5 (8.6) | 14.7 (16) |

Scores represent group mean averages with standard deviation in parentheses
